# Identification of a Plastid-Localized Bifunctional Nerolidol/Linalool Synthase in Relation to Linalool Biosynthesis in Young Grape Berries

**DOI:** 10.3390/ijms151221992

**Published:** 2014-12-01

**Authors:** Bao-Qing Zhu, Jian Cai, Zhi-Qun Wang, Xiao-Qing Xu, Chang-Qing Duan, Qiu-Hong Pan

**Affiliations:** 1 Centre for Viticulture & Enology, College of Food Science and Nutritional Engineering, China Agricultural University, Beijing 100083, China; E-Mails: Zhubaoqing@gmail.com (B.-Q.Z.); caijian928@gmail.com (J.C.); zhiquncn@gmail.com (Z.-Q.W.); xuxiaoqing1988@cau.edu.cn (X.-Q.X.); chqduan@cau.edu.cn (C.-Q.D.); 2 Department of Food Science and Engineering, College of Biological Sciences and Technology, Beijing Forestry University, Beijing 100083, China; 3 Qingdao Huadong Winery Co., Ltd., Northside of Lisha Road, Laoshan District, Qingdao 266000, China

**Keywords:** grape berries, linalool synthase, subcellular localization, gene expression

## Abstract

Monoterpenoids are a diverse class of natural products and contribute to the important varietal aroma of certain *Vitis vinifera* grape cultivars. Among the typical monoterpenoids, linalool exists in almost all grape varieties. A gene coding for a nerolidol/linalool (NES/LINS) synthase was evaluated in the role of linalool biosynthesis in grape berries. Enzyme activity assay of this recombinant protein revealed that it could convert geranyl diphosphate and farnesyl diphosphate into linalool and nerolidol *in vitro*, respectively, and thus it was named VvRILinNer. However, localization experiment showed that this enzyme was only localized to chloroplasts, which indicates that VvRILinNer functions in the linalool production *in vivo*. The patterns of gene expression and linalool accumulation were analyzed in the berries of three grape cultivars (“Riesling”, “Cabernet Sauvignon”, “Gewurztraminer”) with significantly different levels of monoterpenoids. The VvRILinNer was considered to be mainly responsible for the synthesis of linalool at the early developmental stage. This finding has provided us with new knowledge to uncover the complex monoterpene biosynthesis in grapes.

## 1. Introduction

*Vitis vinifera* is an economical and useful grape vine, and its numerous varieties facilitate the making of high-quality wines. Terpenenoids, especially monoterpenoids and sesquiterpenoids, greatly contribute to characteristic varietal aroma of the grapes and wines. Based on the level of free monoterpenoids produced in the berries, Mateo and Jiménez proposed that grape varieties could be generally classified into muscat varieties (up to 6 mg/L of free monoterpenoids), non-muscat aromatic varieties (between 1–4 mg/L) and neutral varieties (less than 1 mg/L) [1]. Typical monoterpenol components of aroma-rich grape varieties are linalool, geraniol, nerol, citronellol and α-terpineol. In particular, linalool is one of the most odour-active odorants [2,3] and has been shown as a crucial constituent of grape berry aroma. For example, in Burgundy region, “Chardonnay 809” is featured “muscat” aroma as a result of its very high concentration of linalool, which is distinguished from “Chardonnay 76” specified as being neutral variety [4]. Linalool also represents the characteristic varietal aroma of non-muscat “Riesling” berries [5,6]. So it is necessary to explore the mechanism of linalool accumulation during the development of grape berries.

Much evidence has demonstrated that higher plants like grape produce terpenenoid precursor, isopentenyl diphosphate (IPP), through two distinct routes: cytosolic mevalonic acid (MVA) pathway and plastidial 2*C*-methyl-d-erythritol 4-phosphate (MEP) pathway [7,8]. The condensation of IPP and its allylic isomer dimethylallyl diphosphate (DMAPP) yields geranyl diphosphate (GPP), farnesyl diphosphate (FPP) and geranylgeranyl diphosphate (GGPP) under the action of GPP synthase, FPP synthase and GGPP synthase, respectively. Afterwards, these products successively convert into different kinds of terpenoids, such as monoterpenes (C10), sesquiterpenes (C15), diterpenes (C20), triterpenes (C30), carotenoids (C40), and so on [8]. In this process, terpene synthase (TPS) plays a critical role in the formation of monoterpenes and sesquiterpenes [9,10]. According to previous reports on phylogenetic analysis of TPS amino acid sequences [11,12,13], TPS family is classified into seven subfamilies designated as TPS-a, TPS-b, TPS-c, TPS-d, TPS-e/f, TPS-g and TPS-h [10]. Among these, the members of TPS-g and TPS-b have been *in vitro* demonstrated to convert GPP, FPP and GGPP into monoterpenes, sesquiterpene and diterpene, respectively [10,13,14,15].

Two natural types of linalool isomers (3*S* and 3*R*) exist in higher plants, while the 3*S*-type are the predominant in grape berries [16]. The genes encoding linalool synthase, have been isolated and characterized from many angiosperms, such as *Clarkia breweri* [17], *Arabidopsis thaliana* [18], rice [19], snapdragon [15], strawberry, poplar and others [20]. According to the stereochemistry of their catalytic products, these known linalool synthases in plants are classified into two groups: 3*S*-linalool synthase (EC 4.2.3.25) and 3*R*-linalool synthase (EC 4.2.3.26). The former one belongs to the TPS-g subfamily, which includes nearly all the group I LIS responsible for the synthesizing 3*S* isomer of linalool. And the latter one belongs to the TPS-b subfamily responsible for the synthesis of 3*R-*linalool.

In *Vitis* *vinifera* groups, multiple terpene synthases (VvTPS) have been functionally identified [14]. The researchers have provided comprehensive annotation of the large *VvTPS* gene family, including chromosomal localization, enzyme functions and phylogeny relevant to the overall plant *TPS* gene family. Several genes of the TPS-g sub family from Pinot Noir and Cabernet Sauvignon have been considered to be potential 3*S*-linalool synthases. *In vitro* experiments showed that VvPNLinNer1, VvPNLinNer2 and VvCSLinNer were bi-functional enzymes and they could convert not only GPP to 3*S*-linalool but also FPP to (*E*)-nerolidol. Similarly, VvPNLNG1-4 also generates 3*S*-linalool, (*E*)-nerolidoland (*E*,*E*)-geranyl linalool when used GPP, FPP and GGPP, respectively, as substrates. However, the *in vivo* role of these enzymes remains unclear. It has generally been accepted that GPP and monoterpenes and triterpene such as 3*S*-linalool and (*E*,*E*)-geranyl linalool are synthesized in plastids, whereas FPP and sesquiterpenes like (*E*)-nerolidol are produced in the cytosol [7,9,21]. In grape berries, stable isotope feeding and tracing experiment also has supported that monoterpenes are synthesized through MEP pathway in plastids [16,22]. Since biosynthetic pathways of monoterpenes and sesquiterpenes are strictly compartmentalized in plant cells [15,21], subcellular localization analysis should be a powerful approach to clearly illustrate real roles of these bi-functional enzymes *in vivo*. However so far there is no report involving subcellular localization of grape bi-functional enzymes related to monoterpene synthesis.

In the present study, a gene encoding a bifunctional linalool/nerolidol synthase was isolated from “Riesling” berries. The biochemical properties of the corresponding enzyme were assessed *in vitro* and its plastic localization was clearly determined to be relevant to linalool synthesis. Additionally, the correspondence analysis was conducted between this gene expression and linalool accumulation in the berries of three cultivars with significantly different monoterpene levels. The result revealed that this enzyme greatly contributed to linalool production at the early stage of berry development. This finding would undoubtedly promote further understanding of the complex monoterpene biosynthesis in grape berry.

## 2. Results and Discussion

### 2.1. Identification of Gene Encoding 3S-Linalool Synthase from Grape Berries

According to the report of Martin *et al.* [14], a total of 32 gene models were annotated as linalool synthase genes or bifunctional linalool/nerolidol synthase in 12× grape genome based on nucleotide sequence similarity. Eighteen gene models out of the above 32 genes were considered to be pseudo genes that might be generated by mutation like nucleotide deletion in the progress of evolution. Only seven full-length cDNA fragments corresponding to six putative gene models (TPS54, 56–58, 61, 63) were screened out from grape cultivars Pinot Noir and Cabernet Sauvignon (PN and CS) cDNA libraries. These fragments coded for VvLinNer, and most of them were from the mixed PN template extracted from stems, berries, flowers and leaves. Here it should be noticed that VvPNLNGl1-4 (corresponding to TPS 57, TPS 63, TPS 58 and TPS 61) all lacked a transit signal peptide at their *N*-terminal. It is suggested that they might only catalyze the generation of nerolidol rather than linalool *in vivo* since sesquiterpenes like nerolidol are synthesized in cytoplasm and monoterpenes like linalool are synthesized in plastid. In the present study, we had been trying to get full-length cDNAs of the TPS54 and TPS56 gene models from “Riesling” berries, but only *VvRiLinNer,* corresponding to TPS56 was successfully obtained from young “Riesling” berries (Table 1). This *VvRiLinNer* sequence (accession numbers: JQ062931) contained an open reading frame (ORF) of 1734 base pairs for a predicted protein of 577 amino acids, a molecular weight of 66.1 kDa and a calculated isoelectric point of 6.22 (conducted by NAMAN6.0). Martin *et al.* [14] previously cloned *VvPNLinNer2* (HM807392 in Genbank) from *Vitis vinifera* Pinot Noir and *VvCSLinNer* (HM807393 in Genbank) from *Vitis vinifera* Carbernet Sauvignon based on the designated gene *VvTPS56*. Here, the *VvRiLinNer* fragment obtained shared very high similarity in ORF with *VvPNLinNer2* (99.9%) and *VvCSLinNer* (99.1%). Accordingly, it is suggested that *VvRiLinNer*, *VvCSLinNer*and *VvPNLinNer2* were considered as allele genes of *VvTPS56* (GSVIVT01005272001) distributing in different grape cultivars. However, the length of *VvPNLinNer2* from “Pinot Noir” was 1839 bp, which had an additional 105 nucleotides in its 5'-terminus compared with the sequences of *VvRiLinNer* and *VvCSLinNer*. Several primers were designed on the basis of GSVIVT01005272001 nucleotide sequence to clone this putative 105-nucleotide sequence from *Vitis vinifera* Riesling berries, but no PCR product was attained. Interestingly, the localization of the VvRiLinNer was *in silico* predicted using three different databases (TargetP, PSORT and ChloroP). The results all showed that this enzyme was present in chloroplast, which is totally the same as VvPNLinNer2 with an additional 105 bp sequence. This phenomenon is difficult to explain at present.

**Table 1:** Annotation of the three terpenoids synthase (*VvTPS*) genes.

GenBank Number	Gene ID (12×)	*VvTPS* Gene Model ^†^	Cloned Genes	Note ^‡^
XM002270140.1	GSVIVT01005272001	*VvTPS56*	*VvRiLinNer* (JQ062931, this study) *VvPNLinNer2* (HM807392) *VvCSLinNer* (HM807393)	Full
XM002270071.1	GSVIVT01005271001	*VvTPS65*	No gene has been cloned	Stop in first exon
XM002266813.1	GSVIVT01005221001	*VvTPS54*	*VvPNLinNer1*(HM807391)	Full

**^†^** and **^‡^**, The “*VvTPS* Gene Model” and “Note” are annotated by Marin *et al.* [14].

**Figure 1:**
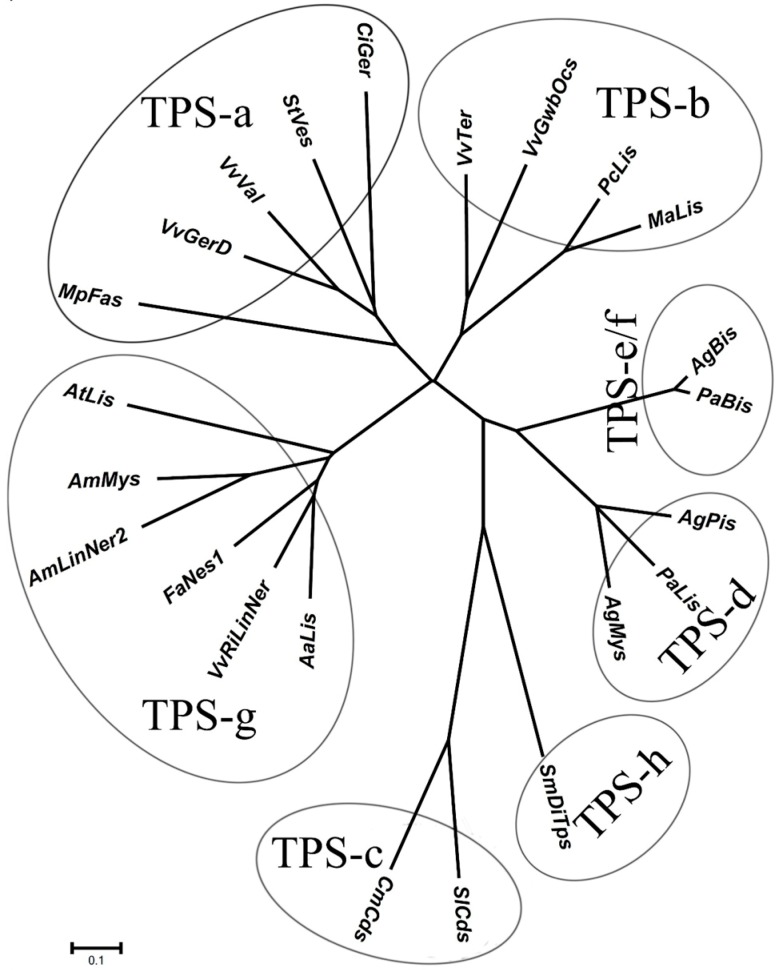
Phylogenetic tree illustrating the VvRiLinNer as a member of terpene synthase (TPS)-g subfamily. The unrooted neighbor-joining tree was created using ClustalX and visualised in TreeView. Division into subfamilies is based on cluster analysis (Bohlmann *et al.* 1998 [11]; Dudareva 2003 [12]; Chen *et al.*, 2011 [10]). Only a subset of the known plant TPSs is shown. Sequence abbreviations: AaLis, *Actinidia argute* linalool synthase (ADD81294); AgBis, *Abies grandis* (*E*)-α-bisabolene synthase; AgMys, *Abies grandis*, myrcene synthase (AAB71084); AgPis, *Abies grandis*, pinene synthase (AAB71085); AmLinNer2, *Antirrhinum majus* linalool/nerolidol synthase (ABR24418); AmMys, *Antirrhinum majus*myrcene synthase (AAO41726); AtLis, *Arabidopsis thaliana* linalool synthase (AAO85533); CiGer, *Cichorium intybus* germacrene A synthase (AAM21658); CmCds, *Cucurbita maxima* copalyl diphosphate synthase (AAD04292); FaNes1, *Fragaria* × *ananassa* nerolidol synthase1 (CAD57083); MaLis, *Mentha aquatic* linalool synthase (AAL99381); MpFas, *Mentha* × *piperita* (*E*)-β-farnesene synthase (AAB95209); PaBis, *Picea abies* (*E*)-α-bisabolene synthase (AAS47689); PaLis, *Picea abies* (−)-linalool synthase (AAS47693); PcLis, *Perilla citriodora* linalool synthase (AAX16075); SlCds, *Solanum lycopersicum* copalyl diphosphate synthase (BAA84918); SmDiTPS, *Selaginella moellendorffii* a bifunctionalditerpene synthase (AEK75338); StVes, *Solanum tuberosum* vetispiradiene synthase (BAA82092); VvGerD, *Vitis vinifera* (−)-germacrene D synthase (AAS66357); VvGwbOcs, *Vitis vinifera* L. Gewürztraminer (*E*)-beta-ocimene synthase (ADR74204); VvRiLinNer, *Vitis vinifera* L. Riesling linalool/nerolidol synthase (JQ062931); VvTer, *Vitis vinifera* (−)-α-terpineol synthase (AAS79352); VvVal, *Vitis vinifera* (+)-valencene synthase (AAS66358).

**Figure 2:**
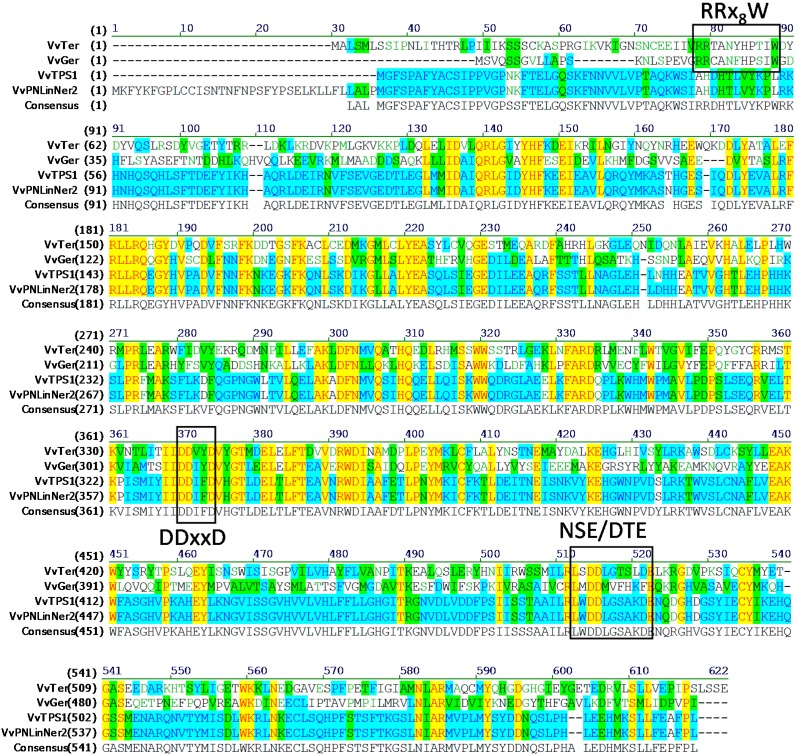
Alignment of the amino acid sequence of VvRiLinNer with other VvTPS. The alignment of VvRiLinNer predicted amino acid sequence with other VvTPS was performed using Align X (Vector NTI Advance 11, Invitrogen). VvTer (GenBank No. AAS79351): (−)-α-terpineol synthase (Martin and Bohlmann 2004 [25]); VvGerD (GenBank No.Q6Q3H3): (−)-germacrene D synthase (Lücker *et al*. 2004 [26]); VvPNLinNer2 (GenBank No. HM807392): linalool/nerlidol synthase, VvCSLinNer (GenBank No. HM807393): linalool/nerlidol synthase (Martin *et al*. 2010 [14]). Letters in different colors or background colors indicate different degrees of sequence similarity between. Following are the explanations in detail. Letters in black font without the background: nonsimilar; green font without the background: weakly similar; green background: block of similar; blue background: conservative; yellow background: identical.

Here it was worthwhile to note that the VvRiLinNer exhibited only approximately 55% and 54% amino acid homology with *Antirrhinum majus* nerolidol/linalool synthases [15], approximately 48% with *Arabidopsis thaliana* linalool synthase [18], as well as about 55% with *Actinidia arguta* linalool synthase [23]. Based on putative amino acid sequences, the VvRiLinNer was grouped into the TPS-g subfamily (Figure 1), which lacks the RRx_8_W motif in the *N*-terminal domain, a key feature of all monoterpene synthases of the TPS-b and TPS-d groups [11,24]. In the *C*-terminal domain, the VvRiLinNer contained two highly-conserved aspartate-rich motifs, DDxxD and NSE/DTE that are functional in the metal-dependent ionization of prenyl diphosphate substrates (Figure 2). The VvRiLinNer also showed the same feature sequences as VvPNLinNer2 and VvCSLinNer (Figure 2). This homology analysis strongly supports this view that the VvRiLinNer performs almost the same biological function as VvPNLinNer2 and VvCSLinNer in grape berry.

**Figure 3:**
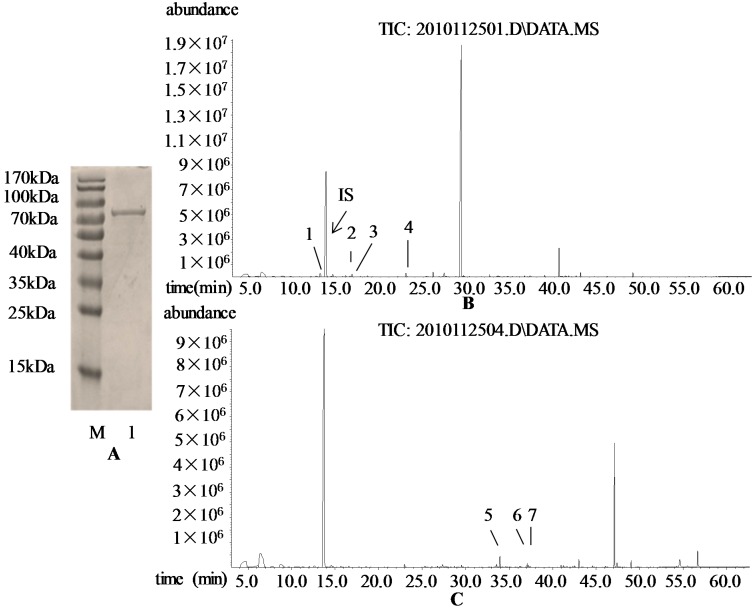
Analysis of SDS polyacrylamide gels of the purified recombinant VvRiLinNer proteins (**A**) and GC-MS analysis of the products of recombinant VvRiLinNer reaction system (**B**,**C**). In (**A**), M: Protein Molecular Weight Marker; 1: the purified protein VvRiLinNer. The apparent molecular weight of expressed recombinant proteins VvRiLinNer was estimated approximately 82 kDa, which is bigger than the predicted proteins from cDNA sequences because of the presence of the His-Tag (approximately 16 kDa); In (**B**) and (**C**), the reaction system contained the prokaryotically-expressed VvRiLinNer and geranyl diphosphate (**B**) or farnesyl diphosphate (**C**). The reaction product (3*S*)-linalool or (*E*)-neroidol was authentically identified. Compounds marked in numbers of (**B**) and (**C**) are listed as follows: 1 β-myrcene, 2 (*Z*)-β-ocimene, 3 (*E*)-β-ocimene, 4 alloocimene, 5 β-farnesene, 6 α-bisabolol, 7 α-farnesene. Among them, the compounds one & five were authentically identified, the others were tentatively identified according to MS spectra comparison with NIST 05 library.

To investigate the catalytic properties of the VvRiLinNer protein, we expressed *VvRiLinNer* in *E. coil* cells and purified the recombinant protein (Figure 3A). *In vitro* reaction analysis showed that VvRiLinNer could convert GPP into (3*S*)-linalool and convert FPP into (*E*)-nerolidol (Figure 3B,C). This means that VvRiLinNer is a bi-functional enzyme *in vitro*, which is similar to VvPNLinNer2 and VvCSLinNer [14]. In addition, we also detected trace levels of β-myrcene, (*E*)-β-ocimene, (*Z*)-β-ocimene and alloocimene in the GPP-containing reaction system, as well as trace levels of α-bisabolol, α-farnesene and β-farnesene in the FPP-containing reaction system (Figure 3B,C). No monoterpene or sesquiterpene was detected in two controls.

### 2.2. Subcellular Localization of VvRiLinNer Protein

**Figure 4:**
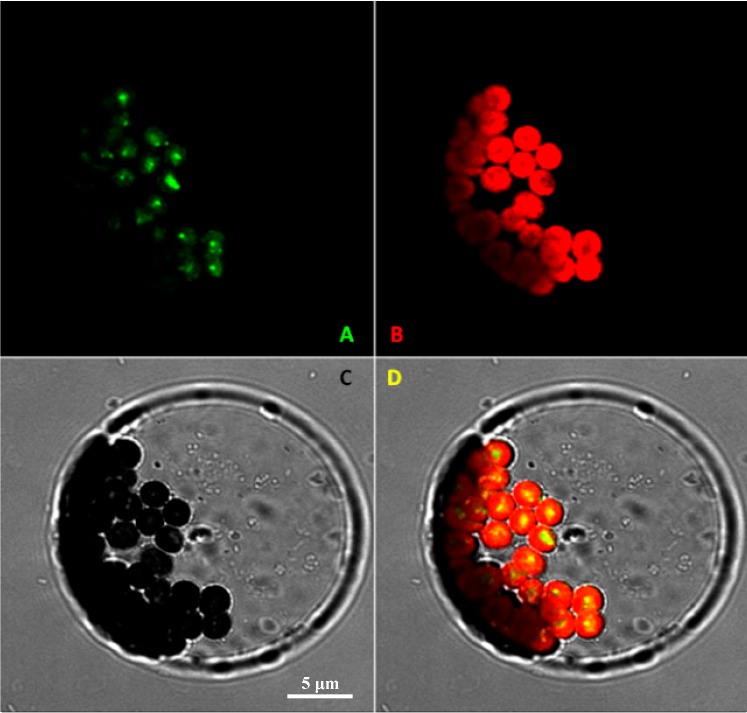
Confocal laser scanning microscopy of transiently expressed VvRiLinNer-GFP fusions in *Arabidopsis* protoplasts (**A**) Green fluorescent protein fluorescence of VvRiLinNer-GFP; (**B**) Chlorophyll autofluorescence; (**C**) Light-microscopy image of intact protoplast. Bar: 5 μm; (**D**) Merged images. All images were taken with the 40× water-immersion objectives on the Zeiss LSM 510 (Zeiss, Germany).

Differential subcellular compartmentalization for the biosynthesis of FPP and GPP (the *in vitro* substrates of VvRiLinNerin plastids and the cytosol, respectively) was found in most plants including grape [8,9,21,27]. To determine *in vivo* localization of this enzyme, we examined the subcellular localization of the VvRiLinNer through transient expression of *VvRiLinNer* fused with a *GFP* reporter gene in *Arabidopsis* protoplasts. GFP fluorescence was clearly restricted in the chloroplasts, and no fluorescence signal was observed in the cytoplasm (Figure 4), which means that the present VvRiLinNer is mainly responsible for the synthesis of 3*S*-linalool *in vivo*. This inference was made mainly from two aspects of evidence. Firstly, using intact plastids separated from cell suspensions of *Vitis vinifera* Muscat de Frontignan, the authors have found that this organelle exhibits the activity of VvGPPS responsible for the synthesis of monoterpene precursor GPP [27]. Secondly, although FPPS and trace FPP pool in wild tomato also have been found to be chloroplast-localized [21,28], the feeding experiment in grape berries with the deuterium labeled precursors [5,5-H-2(2)]-1-deoxy-d-xylulose and [5,5-H-2(2)]-mevalonic acid lactone, a cytosolic MVA pathway-specific precursor, has recently demonstrated that adding IPP concentration would promote more production of sesquiterpenes, but hardly alter level of monoterpenes [22]. IPP is produced from the cytosolic MVA pathway. Up to date, there is still no evidence on presence of FPPS or FPP pool in the plastid of grape berry. Moreover, VvFPPS lacks a plastid signal peptide at *N*-terminal [29]. Combined the present subcellular localization with previous reports, we inferred that the VvRiLinNer acquired in this study should not participate in the synthesis of sesquiterpenes in grape berry.

### 2.3. Correspondence Relationship between VvRiLinNer Expression and Linalool Accumulation in Three Grape Cultivars

**Figure 5:**
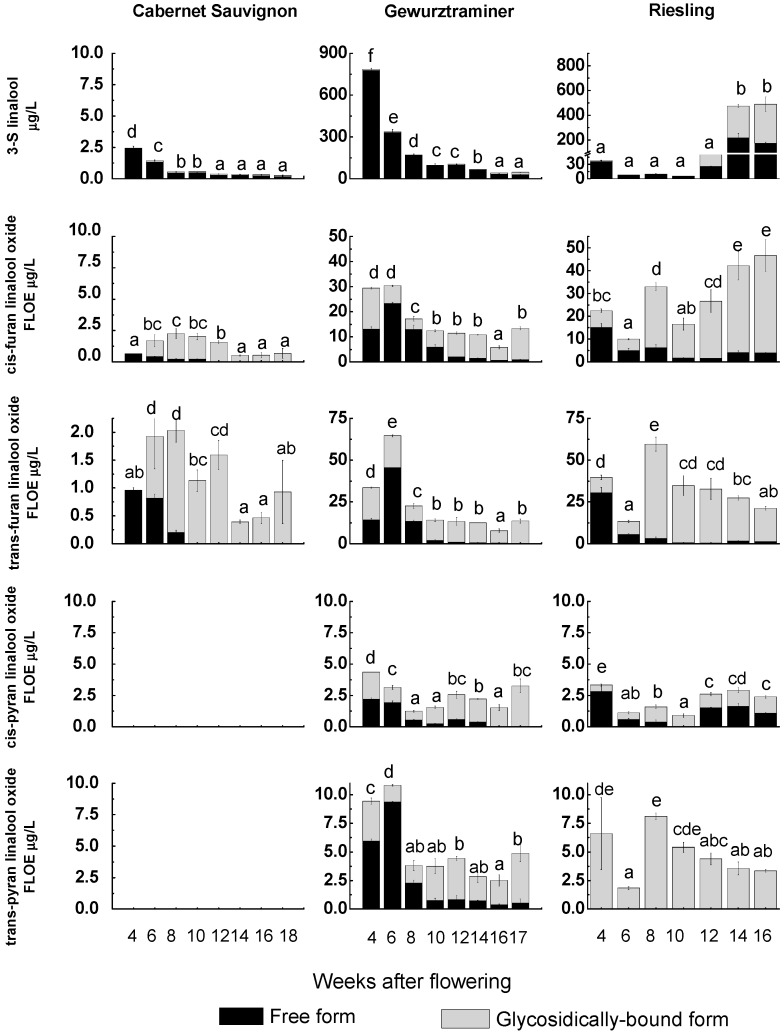
Profiling of linalool and its related oxide in developing grape berries. Each data point is the mean of three values and their standard deviation. Different letters (a–f) indicate that the concentrations of the compound had significantly difference at *p* < 0.05 according to Duncan’s multiple range test in SPSS. Furan linalool oxide equivalents (FLOE).

To further determine the role of the VvRiLinNer in linalool accumulation during grape berry development, we examined free and glycosidically-bound forms of linalool and its structure-linked oxides in three grape cultivars containing Cabernet Sauvignon (CS, a neutral cultivar), Gewurztraminer (Gw, a muscat cultivar) and Riesling (a non-muscat aromatic cultivar) (Figure 5).

Linalool showed an obvious decreasing trend during the development of both CS and Gw berries and existed predominantly in free form. At each development stage, the total concentration of free and glycosidically-bound 3*S*-linalool in Gw berries was always much higher than that in CS berries, a neutral grape cultivar, which was in good agreement with previous observations [2,30,31]. 3*S*-Linalool in young Riesling berries (4 Weeks after flowering “WAF” to 10 “WAF”) was mostly present in free form and declined in the concentration, which was similar to that in Gw and CS varieties. However, a sharp increase in the level of 3*S*-linalool appeared from 12 WAF to 14 WAF. This accumulation pattern was also observed in several muscat vaireties, such as Muscat Hamburg, Moscato Bianco and Muscat de Frontignan [2,31,32,33,34]. This high level was kept till technological harvest (16 WAF). Furthermore, the mature Riesling berries had almost equal contents of free and glycosidically-bound 3*S*-linalool, which was significantly different from Gw and CS berries.

Four linalool oxides were identified from Gw and Riesling berries, including *trans* (*cis*)-furan linalool oxides and *trans* (*cis*)-pyran linalool oxides, whereas only two furan forms were found in CS berries. These linalool oxides existed as both free form and glycosidically-bound form in the grape cultivars studied, apart from only bound-form *trans*-pyran linalool oxide detected in the Reisling berries. For most of the individual linalool oxide, free form generally accounted for higher proportion in young berries and glycosidically-bound form took up higher proportion in mature berries. The concentration of *trans*- and *cis*-furan linalool oxides in CS berries was much lower than those in other two cultivars. In CS and Gw cultivars, total concentration of individual linalool oxide (free form plus glycosidically-bound form, “furan linalool oxide equivalents”) in young berries were generally higher than those in mature berries. The concentration of the four glycosidically-bound linalool oxides (FLOE) all showed a significant increase from eight WAF to 10 WAF and then slightly fluctuated till mature in Gw berries, which is similar to Martin *et al.*’s previous study [29]. Meanwhile the free-type linalool oxides and 3-*S*-linalool kept decreasing. Regarding to “Riesling” cultivar, the concentrations of various linalool oxides in the four WAF berries were all higher than those in the six WAF berries, and at eight WAF these compounds increased with different magnitudes and total concentration of these linalool oxides (FLOE) showed a high level in mature berries. Generally, the overall levels of monoterpenes increase during grape maturation in muscat grape varieties [31,32]. In Muscat Hamburg berries, the main free monoterpenes including linalool increased from veraison [2], while in Muscat de Frontignan free linalool accumulated two weeks after veraison, reached the peak level two weeks later and then decreased until harvest [33]. However the present study showed a different result, that is, total concentration of linalool and its derivatives (FLOE) presented an overall decline trend along the mature of Gw berries.

**Figure 6:**
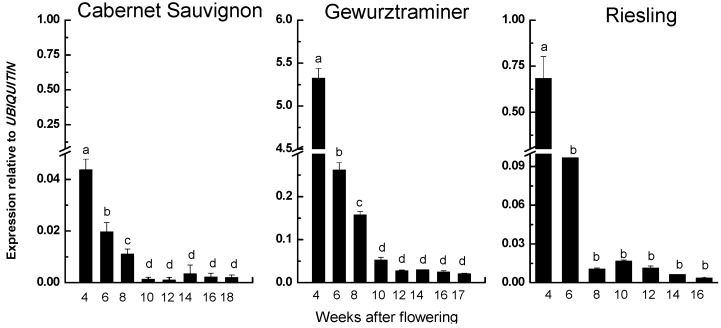
*VvLinNer* relative expression in developing grape berries. Each data point is the mean of six values (multiplying three biological replicates by two technical replicates) and their standard deviation. Different letters (a–d) indicate that the transcript abundance of the gene had significantly difference at *p* < 0.05 according to Duncan’s multiple range test in SPSS.

The transcript abundance of the *VvRiLinNer* was assessed throughout the whole berry development stages. The expression trends of this gene were quite similar in the three grape cultivars (Figure 6). A sharp decrease in expression level was observed from four WAF to eight WAF, which highly positively correlated to the change of total concentration of linalool and its structure-linked compounds. After that, the transcripts of *VvRiLinNer* maintained at low level till berry harvest, which corresponded to low accumulation of linalool in both CS and Gw berries, but was inconsistent with the increase of linalool concentration after veraison in Riesling berries. Based on the above results, we considered that *VvRiLinNer* plays an important role in the synthesis of linalool at early stage of berry development.

The high level of linalool at late developmental stage of Riesling berries could be explained by the existence of other 3*S*-linalool synthases responsible for the production of linalool and its oxides at middle and late developmental stages of “Riesling” berries, as suggested by other researchers. Ebang-Oke *et al.* (2003) observed that a good correlation was observed between the expression of linalool synthase gene (*Lis*) and the accumulation of 3*S*-linalool along with ripening of Muscat de Frontignan grape berries [33]. Matarese *et al.* (2013) recently mentioned that the gene encoding for a bifunctional enzyme VvPNLinNer1 from *Vitis vinifera* Pinot Noir was greatly expressed in ripening Moscato Bianco and Aleatico berries [34].

## 3. Experimental Section

### 3.1. Plant Materials

Grape berries (*Vitis vinifera* L. “Riesling”, “Cabernet Sauvignon”, “Gewurztraminer”) were sampled randomly every two weeks from four weeks after full bloom (WAF) to commercial harvest from 10-year-old grapevines grown in a vineyard located in the suburbs of Beijing, China. Grape berries earlier than four-week were not considered in this study because it was difficult to fully remove the seeds from these small berries. At this location, the commercial harvest date for these three varieties was different: 16 WAF for “Riesling”, 18 WAF for “Cabernet Sauvignon” and 17 WAF for “Gewurztraminer”. At each sampling, about 1000 berries were selected from at least 100-cluster selections at similar position of 50 whole vine selections. Any physically injured, abnormal and infected berries were not selected. The sampling time was fixed at around 10:00 A.M. in the morning. Samples were placed in foam boxes, and transported to the laboratory within two hours. The selected berries were washed with distilled water and dried briefly at room temperature. The seeds of these berries were removed by hand and the remainders were then immediately frozen in liquid nitrogen and stored at −80 °C until use.

### 3.2. Total RNA Extraction, Purification and cDNA Synthesis

The total RNA were extracted from grape berries without seeds by using an improved CTAB (cetyl triethyl ammonium bromide) method [35]. The total RNA were treated with RNase-free DNase to remove grape genome DNA and then purified with the RNA Purification Kit (Bio Basic Inc., Markham, ON, Canada). The quality of the total RNA was verified by demonstration of intact ribosomal bands following agarose gel electrophoresis and the absorbance ratio (A260/A280). For cDNA synthesis, the purified RNA was treated by using Reverse Transcription System Kit (Promega, Madison, WI, USA) and oligo d(T)18 Primer (Takara, Shiga, Japan) according to the supplier’s instruction. The synthesized cDNA was used as template in subsequent study of gene cloning and gene expression. The cDNA from the five-week “Riesling” berries was used to clone the target gene.

### 3.3. Isolation of Linalool Synthase (VvRiLin) Gene from Vitis vinifera Riesling Berries

The gene models which were annotated as putative linalool synthase genes (*LIN*) were obtained from database of VitisNet [36] and FLAGdb++ [37] both based on grape genome [38]. The redundant sequences, together with imperfect models caused by insert/deletion mutation and frame shift, were dropped. The amino acid sequences encoded by the rest of putative full length *LIN* genes were compared with these known plant LINs. Their subcellular localization was predicted through three web tools: ChloroP [39], TargetP [40] and SignalP [41]. The gene models that were predicted to contain a chloroplast-located signal peptide were selected as putative grape linalool synthase genes (in short, *VvLin*s). A blast program was performed to screen DFCI Grape Gene Index database [42] to find EST fragments corresponding to putative *VvLin*s. The *in silico* expression profile of *VvLin*s was analyzed.

Specific primers covering the open reading frame (ORF) of *VvLin* genes were designed with the help of Prime 5.0 software package (Premier Biosoft International, Palo Alto, CA, USA) and were then used to amplify their nucleic acid fragments from grape berry cDNA through polymerase chain reaction (PCR).

The PCR system of a total volume of 20 μL was composed of 1 μL cDNA template, 1 μL of each primer (20 μM), 10 μL 2× Taq PCR plus master mix and 7 μL ddH_2_O (Sinopharm Chemical Reagent Beijing Co., Ltd., Beijing, China) according to the protocol of the supplier with slight modification. The temperature gradient PCR was conducted under various conditions: pre-denaturation at 95 °C for 5 min, denaturation at 94 °C for 30 s, primer annealing at 55 ± 10 °C for 1 min and extension at 72 °C for 1 min 30 s at 35 cycles, a final extension at 72 °C for 10 min, and then cooled to 10 °C in a thermal cycler (Techne TC-512 PCR System, Fisher Scientific UK Ltd., Leicestershire, UK). A band of about 1800 bp was gel-purified, subcloned into a pMD19-T Easy vector (Takara, Shiga, Japan) and sequenced using ABI3700 DNA sequencer (Applied Biosystems, Foster City, CA, USA).

### 3.4. Prokaryotic Expression of VvRiLin and Purification of Recombinant Protein

To obtain prokaryotically-expressed protein, we subcloned the fragment containing *VvRiLin* into a pET32a expression vector (Novagen, Madison, WI, USA). Besides, the corresponding ORF was amplified from the reconstructive plasmid pMD19T-*VvRiLin* by PCR using Pfu DNA polymerase. Forward primer used was 5'-CGGAATTCATGGGATTCTCTCTTGCCTTTTACGC-3' containing an *EcoR* I site (underlined) at the initiating ATG codon. Reverse primer was 5'-CTTGACGCGTCGACCTACAGGGGAAATGCTTCAAAG-3' including a *Sal* I site (underlined). The encoding region of *VvRiLin* amplified by PCR was subcloned into the pET32a expression vector downstream of the (His)_6_ tag. The reconstructive plasmid pET32a-*VvRiLin* was transformed into *E. coli* Rosetta (DE3). A plasmid DNA was purified and verified by sequencing. Single colonies of *E. coli* Rosetta (DE3) with pET32a-*VvRiLin* were incubated overnight at 37 °C and 180 rpm in a 5-mL Luria-Bertani medium supplemented with ampicillin (100 μg/mL). After that, the medium of 500 μL was transferred into a 500-mL volume of fresh Lysogeny broth (LB) medium containing ampicillin (100 μg/mL). The bacterial culture was kept shaking in a water bath at 37 °C until reaching an optical density of 0.8 at a wave length of 600 nm. Isopropyl-β-d-thiogalactoside (IPTG) was then added into this medium to a final concentration of 1 mM. The medium was incubated at 16 °C and 150 rpm for another 16 h to reach an optimal protein concentration. The *E. coli* cells containing target protein were collected by centrifugation and further subjected to a purification procedure using Ni-NTA His Band Resin (Novagen, Madison, WI, USA). The purified protein was dialyzed against a buffer (30 mM HEPES, pH 7.2, 7.5 mM MgCl_2_, 20 μM MnCl_2_, 5% glycerol, 5 mM DTT) [25] as described by Fäldt *et al.* [43]. The analysis of SDS-Polyacrylamide gel electrophoresis showed that there existed a single band corresponding to the target protein on a 10% polyacrylamide gel (Biodee Biotechnology Co., Ltd., Beijing, China), which indicated that the prokaryotically-expressed protein obtained could meet the requirement of identification of biochemical function.

### 3.5. Catalytic Activity of the Purified VvRiLinNer Protein

Catalytic activity of the prokaryotically-expressed protein was performed in a 500-μL reaction buffer (pH 7.2) containing 30 mM HEPES (Biodee Biotechnology Co., Ltd., Beijing, China), 7.5 mM MgCl_2_, 20 μM MnCl_2_, 5% glycerol and 5 mM DTT (Sinopharm Chemical Reagent Beijing Co., Ltd., Beijing, China) and recombinant protein (0.5–0.8 μg) and 55/46 μM GPP/FPP (Echelon Bioscience Inc., Salt Lake City, UT, USA). Two types of the control were taken: one was to use the extracts from *E. coli* cells containing pET32a without the target gene instead of the recombinant VvRiLinNer protein and the other to use the heat-denatured recombinant VvRiLinNer protein. After incubation for 1 h at 30 °C, the reaction products were extracted with headspace-solid-phase micro-extraction (HS-SPME), and determined through GC–MS. The HS-SPME procedures were shown as follows: 500 μL reaction mixture, 1 μL 4-methyl-2-pentanol (1.039 mg/L water, internal standard) and 0.20 g NaCl were blended in a 15-mL sample vial tightly capped with a PTFE silicon septum and containing a magnetic stirrer. The vial was equilibrated at 40 °C for 30 min on a heating platform agitation. Then activated SPME fiber (50/30 μm DVB/Carboxen/PDMS, Supelco, Bellefonte, PA, USA; pretreated at 270 °C for 1 h) was inserted into the headspace for 30 min with continued heatingand agitation. Finally, the fiber was instantly desorbed in the GC injector for 8 min. Three replicates were conducted as to each reaction.

Quantitative analysis was performed on an Agilent 6890 Series GC System equipped with an Agilent 5975 Network Mass Selective Detector. The injector port was maintained at 250 °C. Helium (Millennium City Gas Co., Ltd., Beijing, China) was used as the carrier gas at 1 mL/min, and GC inlet was set in the splitless mode. The HP-INNOWAX capillary column (60 m, 0.25 mm ID, 0.25 μm film thickness) was used for peak identification. The procedure was as follows: an initial temperature of 50 °C (1 min hold) was increased to 220 °C at 3 °C/min and held at 220 °C for 5 min. Mass spectra in the electron impact mode at a voltage of 70 eV ionization energy was set to scan from *m*/*z* 20 to 450 and operated in the selective ion mode under autotune conditions. The products were identified through authentic standards or mass spectra matching in the standard NIST05 library.

### 3.6. Subcellular Localization of VvRiLinNer

For the observation of subcellular localization of VvRiLinNer, the open reading frame of *VvRiLinNer* was PCR-amplified with reference to the method described in “Prokaryotic expression of *VvRiLinNer*” section above. Forward primer (5'-CGGAATTCATGGGATTCTCTCCTGCCTT-3') and reverse primer (5'-CGGGATCCAGGGGATATGCTTCAAAGAG-3') were adopted. The amplicon was digested with *EcoR*I/*Hind* III (underlined) restriction enzymes. The PCR product was fused with the upstream of the enhanced GFP in the cauliflower mosaic virus 35S-EGFP-Ocs 3' vector (p-EZS-NL vector; [44]). This p-EZS-NL vector does not express GFP well without adding a coding sequence to the 5' end of the ORF of *GFP*; thus, the control cells do not show fluorescence of GFP [45]. The pEZS-NL vector and the *Arabidopsis* (ecotype Columbia) seeds were kindly provided by Professor Zhang Dapeng (School of Life Sciences, Tsinghua University, Beijing, China). Sequencing analysis confirmed the accuracy of fusion. *Arabidopsis* protoplasts were isolated from the leaves of three- to four-week old plants of *Arabidopsis* and transiently transformed through polyethylene glycol as described by Sheen (2002) [46]. Transient expression of GFP fusion protein was observed with a confocal laser-scanning microscope (LSM 510 META, Zeiss, Germany) after incubation at 23 °C for 16 h.

### 3.7. Analysis of VvRiLinNer Expression by Real Time PCR

Analysis of gene expression was performed by Real time PCR using the SYBR green method on an ABI 7300 Real-Time System (Applied Biosystems, Foster City, CA, USA). RNA isolation and cDNA preparation were conducted as described above. The synthesized cDNA was quantified and all the tested samples were adjusted to the same concentration. Each Real time PCR reaction (30 μL) contained 15 μL SYBR^®^ Premix Ex Taq™, 0.6 μLRox Reference Dye (50×) (TaKaRa , Shiga, Japan), 13.5 μL ddH_2_O, 0.5 μL cDNA and 1 μL primer mixture (forward primers and reverse primers, 10 μM). A 162-bp fragment in the 3' region of the coding sequence was amplified by using specific primers designed by PerlPrimer (http://perlprimer.sourceforge.net/) [47]. The primers used were as follows: forward primer (5'-ATGACAATCAGAGTCTTCCACAC-3') and reverse primer (5'-AAGAAACGAAGAGACAATCCTG-3'). *VvUbiquitin* (GenBank No. AY684131) that is stably expressed in grape berry was selected as the internal control, and primers for it were *Ubiquitin* F (5'-GTGGTATTATTGAGCCATCCTT-3') and *Ubiquitin* R (5'-AACCTCCAATCCAGTCATCTAC-3'). The specification of the primers was verified on the basis of the following two facts: firstly, the dissociation curve for a pair of primers presented a single peak, and secondly, the fragment size of PCR product was consistent with the theoretical prediction, and the fragment sequence was shown to be correct through nucleotide sequencing.

For real time PCR, the template cDNA was denatured at 95 °C for 30 s followed by 40 cycles of amplification with 94 °C for 10 s, 60 °C for 31 s [48]. As to each grape sample, three independent RNA extraction replicates were done and each replicate was performed with two amplification reactions. The average cycle threshold (*C*t) of *VvUbiquitin* at each sampling point was determined and transcript abundance of the target gene, normalized to *VvUbiquitin*, was calculated using the formula 2^−∆*C*t^, where ∆*C*t = *C*t_,target_ – *C*t_,VvUbquitin_. The mean and the standard derivation (SD) were estimated after 2^−∆*C*t^ calculation.

### 3.8. Quantification of Linalool and Its Derivatives by GC–MS

The berry samples without seeds, stored at −80 °C, were crushed and steeped for 140 min at 4 °C, and then were immediately centrifuged at 4 °C and 6000*×* *g* for 10 min. The supernatant was collected. The glycosidically-bound linalool and its related compounds were isolated by the Solid-Phase Extraction (in sort, SPE) method. In short, 5 mL of the clear juice was passed through the Cleanert PEP–SPE column (Bonna-agela Technologies ,Tianjin, China, 200 mg/6 mL), which was activated in advance with 10 mL methanol (Sinopharm Chemical Reagent Beijing Co., Ltd., Beijing, China) and 10 mL water. And then the water-soluble compounds were eluted with 5 mL of water, the free volatiles with 10 mL of dichloromethane (Sinopharm Chemical Reagent Beijing Co., Ltd., Beijing, China) and the target bound volatiles with 20 mL of methanol, sequentially. The flow rate was about 2 mL/min. The methanol elute was evaporated to dryness by reduced pressure distillation at 30 °C and then redissolved in 5 mL citrate-phosphate buffer solution (2 M, pH 5.0). Subsequently, 100 μL of the AR 2000 solution (DSM Food Specialties, Séclin, France, 100 mg/mL) was added to the glycoside extract and the mixture was vortexed and enzymatically hydrolyzed in an incubatorat 40 °C for 16 h to liberate the free aroma according to optimum conditions described previously [49]. The tube containing the mixture was stored at 4 °C and the liberated volatiles were extracted through SPME fiber within two days. Free types of linalool and its related compounds were extracted using HS-SPME according to the method of Wu *et al.* (2009) with minor modification [49]. That is, the grape juice of 5 mL, 10 μL 4-methyl-2-pentanol (1.039 mg/mL water, internal standard, Sigma-Aldrich) and 1.00 g NaCl (Sinopharm Chemical Reagent Beijing Co., Ltd., Beijing, China) were blended in a 15-mL sample vial tightly capped with a PTFE–silicon septum and containing a magnetic stirrer. The procedures of HS-SPME and GC–MS analysis were performed as described in the above section “assay of terpene synthase activity”. As to each sampling point, three independent extraction replicates were conducted.

The quantification procedure used was based on our prior research with some modifications [50]. According to the average concentration of sugar and acids in grape juice, the model solution consisted of 180 g/L glucose and 7 g/L tartaric acid and pH value was adjusted to 3.3 with NaOH (Sinopharm Chemical Reagent Beijing Co., Ltd., Beijing, China). The chemical standards used for quantification were linalool (96.5%, Sigma-Aldrich, Switzerland) and furan linalool oxides (97% purity, mixture of *cis-* and *trans-*furan linalool oxide, Fluka, Japan). Each standard was dissolved with ethanol (HPLC quanlity, Anpel Co., Ltd., Shanghai, China). All the standard stock solutions were then combined together, and eight different and successive concentrations of mixed standard solutions were prepared in juice model solution. Standard solutions of each level was extracted by HS-SPME and analyzed by GC–MS under the same condition as that of grape samples. The calibration curve of linalool was obtained and directly used for linalool quantification. As for furan linalool oxide, the calibration curve was acquired using the sum of two peak areas corresponding to *cis*- and *trans*-isomers, respectively. To quantify either of furan isomers in grape berry, we used this calibration curve to estimate the content of *trans*- or *cis*-furan linalool oxide on the basis of the peak area of single isomer. Also two pyran linalool oxide isomers were quantified using this furan calibration curve because there were no pyran linalool oxide standards bought. Similarly, only the peak area of the corresponding compound was considered when the content of *trans*- or *cis*-pyran linalool oxide was estimated. In this study, both linalool and furan linalool oxide calibration curves had above 99% of regression coefficient.

### 3.9. Statistics Analysis

A one-way analysis of variance (ANOVA) was carried out using the IBM SPPS Statistics 20 software (IBM Corp, Armonk, NY, USA). The statistical significance of the differences in the levels of linalool related compounds or gene expression among the berries of different developmental stages was examined employing Duncan’s multiple range test at *p* < 0.05.

## 4. Conclusions

The present study highlights subcellular localization of the VvRiLinNer from *Vitisvinifera* “Riesling” berry and the role of this enzyme in the synthesis of linalool and its derivatives in three grape cultivars with significantly different monoterpene levels. Combined strict chloroplast localization and previous reports, the VvRiLinNer obtained in this study is determined to be only responsible for linalool synthesis in grape berry although *in vitro* experiment shows that it is a bi-functional enzyme, catalyzing the conversion of GPP into linalool and FPP into nerolidol. Considering all linalool-related components including free and glucosidically-bound linalool as well as their derivatives, a close correlation exists between linalool accumulation and *VvRiLinNer* expression in young grape berries. These observations have added some new knowledgeof the accumulation of a wide verity of terpenes in grape berries. Meanwhile, some new questions arise, for example, what factors regulate the gene expression of *VvRiLinNer* in developing grape berries; what other genes are responsible for the synthesis of linalool and its oxides in middle and late developmental stages of Riesling berries; what differences exist between nucleotide sequences of these unknown genes and *VvRiLinNer,* and so on. Future studies are to be made to solve these problems.
